# Antimicrobial and anti-biofilm efficacy of different inorganic and organic zinc forms against multidrug-resistant *Escherichia, Klebsiella, Staphylococcus* and *Pseudomonas*

**DOI:** 10.1007/s11259-024-10339-7

**Published:** 2024-03-01

**Authors:** Lívia Karahutová, Dobroslava Bujňáková

**Affiliations:** Institute of Animal Physiology, Centre of Biosciences of the Slovak Academy of Sciences, Šoltésovej 4/6, 040 01 Košice, Slovak Republic

**Keywords:** Antimicrobial activity, Anti-biofilm capacity, Multidrug resistant bacteria, Organic zinc, Inorganic zinc

## Abstract

In our study antibacterial and anti-biofilm efficacy of 2 inorganics (Zn(II) sulphate monohydrate; Zn(II) sulphate heptahydrate) and 3 organic Zn(II) substances (Zn(II) chelate of protein hydrolysate: Zn-Bio; Zn(II) chelate of amino acid hydrate: Zn-AMK; Zn(II) chelate of glycine hydrate: Zn-Gly) were explored and compared against multidrug resistant *Escherichia coli (E. coli), Staphylococcus aureus (S. aureus), Klebsiella oxytoca (K. oxytoca)* and *Pseudomonas aeruginosa (P. aeruginosa)* using by the 96- wells microtiter plate-based resazurin and/or crystal violet assay. Our finding confirmed that Zn(II)-sulphates and Zn(II)-amino acid complexes exhibit dose and genus-based antibacterial and anti-biofilm potential. Organic compounds (Zn-AMK and Zn-Gly) were more effective against bacterial growth, except *P. aeruginosa*. Besides Zn-AMK, others organic and inorganic forms of Zn(II) caused predominantly statistically significant decrease of biofilm production in all of tested bacteria. Current data highlights that Zn(II) in various forms has a great potential to be developed as antibacterial and anti-biofilm agents.

## Introduction

Biofilms are estimated to be responsible for more than 65% of nosocomial infections, approximately 80% of chronic infections (e.g. chronic wounds, “staph” skin infections) and 60% of all human bacterial infections (Assefa and Amare 2022) and what’s more, biofilms can impact antimicrobial efficacy and contributing to antimicrobial resistance. The therapy of biofilm-related multidrug-resistant bacteria has been shown often to be ineffective, since many agents are unable to achieve the target bacteria mounted deep inside the biofilm matrix. Therefore, an alternative approach is needed to control the diseases involving multidrug resistant bacteria in biofilms (LewisOscar et al. 2015). Different metals with the ability to target multiple sites in an organism make them better to conventional antibiotics (Sondi and Salopek-Sondi 2004). Metal ions are absorbed through the cell membrane, followed by direct interaction with the functional groups of proteins and nucleic acids, such as mercapto (–SH), amino (–NH), and carboxyl (–COOH) groups, damaging enzyme activity, changing the cell structure, affecting the normal physiological processes, and ultimately inhibiting the microorganism. Different metal ions have different sites of activity; for example, zinc ions can bind with high affinity to the –SH groups of proteins. The ordered and closely spaced cell membranes become confused and dispersed, destroying their inherent function and leading to bacterial death (Wang et al. 2017).

Though the precise mechanisms of metal compounds remain largely unclear at present, the generation of reactive oxygen species may account for a possible mechanism for antibacterial activity, which has been reported by many researchers until now (Wang et al. 2019; Zoltan et al. 2015).

Various zinc derivatives are often utilized in different areas of medicine (dermatology, dentistry), in pharmaceutical, cosmetic and food industry to control microbial growth and biofilm. However, due to the frequent use zinc-containing antibiotics are becoming insufficient due to increasing bacterial resistance. Therefore, the search for new zinc (Zn) antimicrobials is still a great challenge (Abendrot and Kalinowska 2018). Previous in vivo studies demonstrated many beneficial impacts of supplemental Zn (organic or inorganic) on different physiological and immunological functions, positive impact on animal health and performance, and some of them deal with the effect on the gut bacterial composition (Bujňáková et al. 2023), since Zn in appropriate concentration is essential for microorganisms, although conversely, the antimicrobial effect of excess Zn is well-known (Molnar-Nagy et al. 2022). The most common used inorganic Zn derivatives are Zn(II) sulphates. However, they have been found to possess low bioavailability in organisms, but in contrast, results of several in vitro studies showed that the Zn(II) sulphate compounds have a higher potential to inhibit the bacterial growth and these results indicate that Zn(II) from inorganic Zn sources is toxic to microorganisms. Thus as a safer alternative are indicated candidates comprises Zn compounds containing organic moieties, which in general have shown better tolerability and bioavailability than the inorganic salts (Abendrot et al. 2020; Sobel and Theophall 2010). However, generally little is known about the effects of different forms and quantities of Zn on the various bacteria and simultaneously although numerous reports have analysed the anti-bacterial effect of various Zn substances, limited studies have evaluated the inhibitory effect of Zn on biofilm formation.

In the present study, measurements regarding minimum inhibitory concentration (MIC) values of water-soluble organic and inorganic Zn sources were performed to better understand which Zn compounds or concentrations could stimulate or inhibit the microbial growth and biofilm of several representative Gram-positive or Gram-negative bacteria, be more specific *Escherichia coli (E. coli), Staphylococcus aureus (S. aureus), Klebsiella oxytoca (K. oxytoca)* and *Pseudomonas aeruginosa (P. aeruginosa)*.

## Materials and methods

### Bacterial isolates

The multi-resistant bacteria were collected from various sources. Briefly, *E. coli* isolate was from the chicken cloacal swab with enteritis, *S. aureus* from human skin lesion, *K. oxytoca* from oral human cavity and *P. aeruginosa* from bovine lungs. Strains were identified using by MALDI-ToF MS (Matrix-Assisted Laser Desorption/Ionization – Time of Flight Mass Spectrophotometry) biotyper (Bruker Daltonics, Bremen, GER). The isolates showed antimicrobial multi-resistance profile (resistant to at the least three different classes of antibiotics), be more specific: *E. coli* – ampicillin, ampicillin + sulbactam, cefotaxime, ceftazidime, tigecycline, ciprofloxacin, tetracycline, trimethoprim + sulphonamide; *S. aureus* –ampicillin, oxacillin, cefoxitin, chloramphenicol, tetracycline, trimethoprim + sulphonamide; *K. oxytoca* – ampicillin, ampicillin + sulbactam, cefuroxim, ciprofloxacin, tetracycline, trimethoprim + sulphonamide and *P. aeruginosa* – ampicillin + sulbactam; ciprofloxacin, tetracycline and trimethoprim + sulphonamide.

### Zn inorganic and organic substances

For testing were used:

1. Zn(II) sulphate monohydrate (ZnSO_4_; Mr = 179.5 g/mol), (Sigma Aldrich, Saint Louis, USA)
2. Zn(II) sulphate heptahydrate (ZnSO_4_ × 7 H_2_O; Mr = 287.56 g/mol), (Sigma Aldrich, Saint Louis, USA)
3. Zn(II) chelate of protein hydrolysate (Bioplex) – Zn-Bio, (ALLTECH Inc., Kentucky USA)
4. Zn(II) chelate of amino acid hydrate (Availa) – Zn-AMK, (Zinpro Animal Nutrition, BOXMEER, Netherlands)
5. Zn(II) chelate of glycine hydrate (Glycinoplex) – Zn-Gly, (PHYTOBIOTICS, Eltville, Germany)

### Antibacterial and anti-biofilm activities of Zn inorganic and organic substances

The efficiency of different Zn substances on bacterial growth was investigated by the 96- wells microtiter plate resazurin-based methods according Sarker et al. (2007) with small modifications.

Stock concentrations of Zn substances (ZnSO_4_ = 8 mg/mL; ZnSO_4_ × 7 H_2_O = 12 mg/mL; Zn-Bio = 10 mg/mL; Zn-AMK = 12 mg/mL; Zn-Gly = 12 mg/mL) was diluted in sterile water and two-fold serial dilution was prepared using by specific broths [Muller- Hinton (MH, Oxoid, Basingstoke, Hampshire, UK) for *E. coli* and *K. oxytoca*; and for *S. aureus* and *P. aeruginosa* Brain Heart infusion (BHI, Oxoid, Basingstoke, Hampshire, UK)]. Then, the resazurin indicator and bacterial suspension in final concentration of 0.5 McFarland was added. Each plate had a positive (bacteria in broth without Zn substances) and negative (solution without bacteria with Zn substances) controls. The plates were incubated for 24 h at 37 °C. The evaluation of the Zn substances effects to bacterial growth were assessed visually according to Sarker et al. (2007).

Testing of Zn substances to biofilm formation was determined according to previously reported method (Bujňáková and Kmeť 2012) with some modifications. Briefly, individual bacteria were resuspended in Phosphate-Buffered Saline (PBS; Oxoid, Basingstoke, Hampshire, UK) to reach the McFarland standard 1 suspension and were administered into 96- well microtiter plates (Thermo Scientific™, Roskilde, DNK) without (positive control) or with various concentration of Zn substances [ZnSO_4_ and ZnSO_4_ × 7 H_2_O, (0. 25–6 mg/mL); Zn-AMK, (0.375–10 mg/mL); Zn-Gly, (0.18–10 mg/mL); Zn-Bio, (0.03–8 mg/mL)]; afterwards, microtiter plates were incubated at 37 °C for 48 h. Then wells were stained with a 1% (W/V) solution of crystal violet (Mikrochem, Pezinok, SVK), washed with distilled water, drying and 200 µL of the ethanol–acetone (80:20, V/V) mixture was added. The absorbance at 570 nm of the dye solutions was measured in Synergy HT Multi-Mode Microplate Reader (BioTek, Vermont, USA). The inhibitory effect of the various Zn solutions on bacterial biofilms was calculated using following formula:

$${\text{Inhibition}}\left( \% \right) = \frac{{{\text{A}}\,{\text{of}}\,{\text{untreated}}\,{\text{control}} - {\text{A}}\,{\text{of}}\,{\text{treated}}\,{\text{sample}}}}{{{\text{A}}\,{\text{of}}\,{\text{untreated}}\,{\text{control}}\, \times \,100}}$$

where A is absorbance measured at 570 nm.

The % of residual adhesion after treatment with various Zn substances was calculated using following formula:

$${\text{Residual}}\,{\text{adherence}}\left( \% \right) = 100\left( \% \right)\,{\text{ - }}\,{\text{inhibition}}\left( \% \right)$$

### Statistical analysis

The experimental data were analysed using GraphPad Prism version 5 (GraphPad Software, Boston, MA) with ANOVA one-way analysis of variance and Dunnett’s Multiple Comparison Test, values of *p* ≤ 0.05 were considered as significant.

## Results

### Antibacterial and anti-biofilm activities of Zn inorganic and organic substances

The results of antibacterial activity as shown in Table 1, demonstrate that effective doses for various Zn substances for both Gram-positive and/or Gram-negative bacteria are different.

**Table 1:** Minimum inhibitory concentration (MIC) values of Zn complexes for Gram-negative and Gram-positive bacteria

Microorganisms	MIC of active substances (mg/mL)				
	ZnSO_4_	ZnSO_4_ × 7 H_2_O	Zn-AMK	Zn-Gly	Zn-Bio
*E. coli*	2	6	1.5	0.75	0.125
*K. oxytoca*	2	3	3	1.5	8
*P. aeruginosa*	2	2	10	10	8
*S. aureus*	1	3	1.5	3	4

Abbreviations: Zinc sulphate monohydrate (ZnSO_4_), Zinc sulphate heptahydrate (ZnSO_4_ × 7 H_2_O), Zinc chelate of glycine hydrate (Glycinoplex) – Zn−Gly, Zinc chelate of amino acid hydrate (Availa) – Zn−AMK and Zinc chelate of protein hydrolysate (Bioplex) – Zn−Bio.

MIC values of both ZnSO_4_ against *E. coli, K. oxytoca* and *P. aeruginosa* ranging from 2 to 6 mg/mL and for *S. aureus* MICs were a little lower and moving from 1 to 3 mg/mL. The MIC values of organic moieties moved from 0.125 to 10 mg/mL for all examined bacteria. Obtained records revealed that organic complexes in *E. coli* demonstrate generally higher effect on bacterial growth in comparison with inorganic ZnSO_4_. Complex Zn-Bio possess the highest antibacterial properties, its MIC value was found to be 0.125 mg/mL, followed by Zn-Gly with MIC ═ 0.75 mg/mL and Zn-AMK with MIC ═ 1.5 mg/mL for *E. coli.* Similar results of Zn-Gly were obtained also in case of *K. oxytoca* (MIC ═ 1.5 mg/mL). Others two organic moieties had comparable or little bit higher antibacterial properties as inorganic substances for *Klebsiella* (Zn-AMK, MIC ═ 3 mg/mL; Zn-Bio, MIC ═ 8 mg/mL in comparison with ZnSO_4_, MIC ═ 2 or ZnSO_4_ × 7 H_2_O, MIC ═ 3 mg/mL). Although organic compounds were generally more effective against bacterial growth, *P. aeruginosa* isolate was an exception. MICs were ranged at higher levels from 8 to 10 mg/mL. Both inorganic ZnSO_4_ substances demonstrated better antibacterial activities against *P. aeruginosa* and *S. aureus* e contra organic complexes however an almost comparable effect was seen in *S. aureus* after application of Zn-AMK and ZnSO_4_ (MIC ═ 1.5 mg/mL vs. MIC ═ 1 mg/mL) and Zn-Gly with ZnSO_4_ × 7 H_2_O (in both cases MIC ═ 3 mg/mL).

**Fig. 1:**
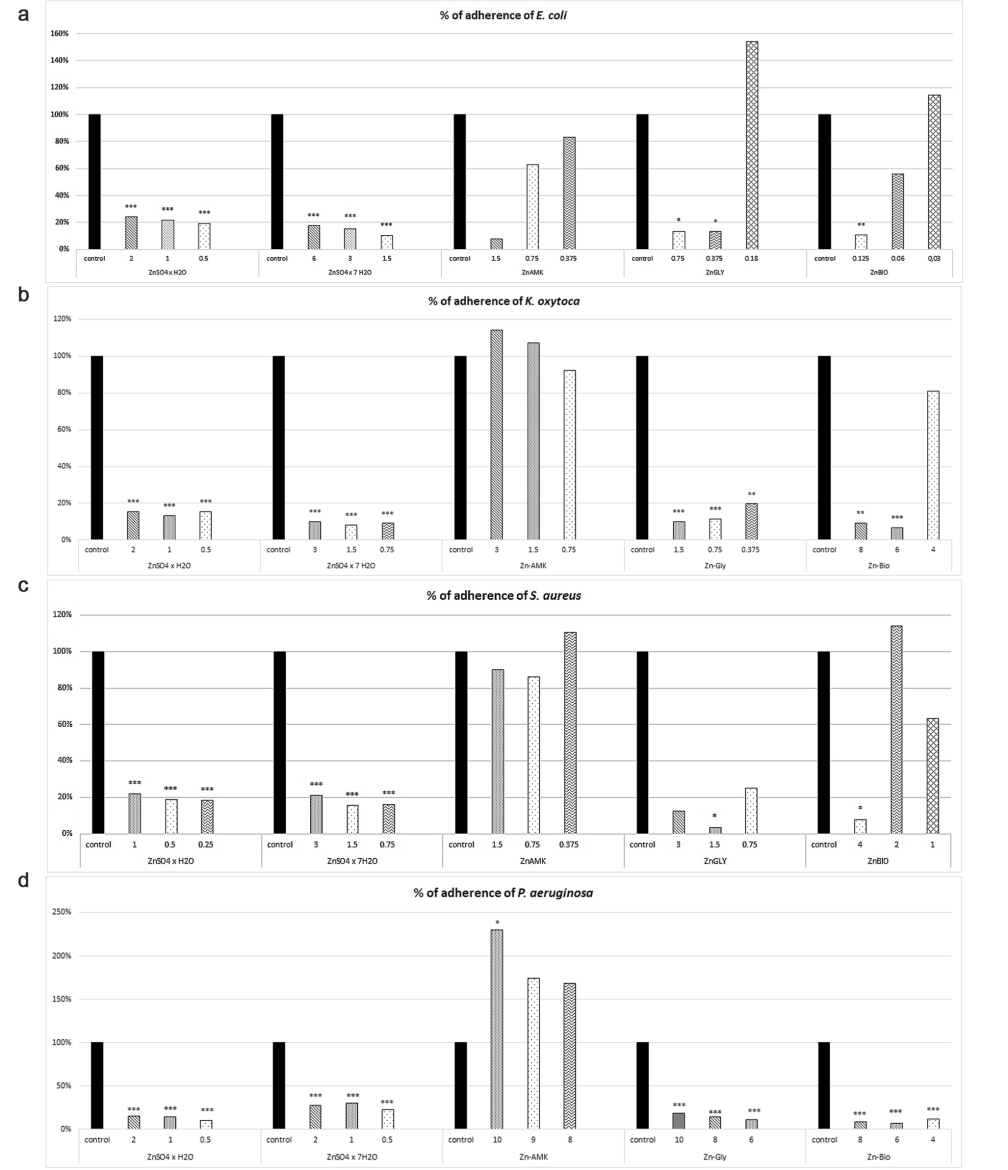
Percentage of bacterial adherence in biofilm formation after treatment with different concentrations of various Zn complexes; **(a)*** E. coli*, **(b)*** K. oxytoca*, **(c)*** S. aureus* and **(d)*** P. aeruginosa*. Abbreviations: Zinc sulphate monohydrate (ZnSO_4_), Zinc sulphate heptahydrate (ZnSO_4_ x 7 H_2_O), Zinc chelate of amino acid hydrate (Availa)- Zn−AMK, Zinc chelate of glycine hydrate (Glycinoplex), Zn−Gly and Zinc chelate of protein hydrolysate (Bioplex)–Zn−Bio

The results of anti-biofilm activity of the 3 organic and 2 inorganic complexes toward selected bacteria are illustrated in Fig. 1a-d.

Inorganic ZnSO_4_ monohydrate (concentration spectra 0.25–2 mg/mL; with MIC value 1 or 2 mg/mL, depending on the species) possesses in all tested isolates statistically significant relatively high inhibition of biofilm formation in the range from 75 to 80% in *E. coli*, around 85% in *K. oxytoca*, 78 − 82% in *S. aureus* and 85–90% in *P. aeruginosa*. Similar results, biofilm inhibition until 89% were obtained with ZnSO_4_ × 7H_2_O in *E. coli*, around 91% in *K. oxytoca*, 84% in *S. aureus* and 59 − 77% in *P. aeruginosa* at concentrations ranging from 1.5 to 6 mg/mL (Fig. 1a and d).

Besides Zn-AMK others organic forms of Zn caused predominantly statistically significant decrease of biofilm production – Zn-Gly (concentration from 0.375 to 10 mg/mL) in all of tested bacteria (range for *E. coli* − 86%, for *K. oxytoca* 80–90%, *S. aureus* 96% and *P. aeruginosa* 81 − 89%), except for some cases where the biofilm increased but insignificantly (Fig. 1a and d). Zn-Bio showed significant decrease in all tested concentrations (4–8 mg/mL) in *P. aeruginosa* (88 − 92%), in two concentrations (6 and 8 mg/mL) in *K. oxytoca* (around 90%), 88% for *E. coli* (0.125 mg/mL) and 92% for *S. aureus* (4 mg/mL).

## Discussions

### Antibacterial and anti-biofilm activity of zn(II) inorganic and organic substances

The inappropriate use of antibiotics has resulted in the emergence of multidrug-resistant biofilm forming bacteria, therefore, a great number of studies have focused on the search for newer, more effective antimicrobial and anti-biofilm agents (Sharma et al. 2022). Literature data indicate some transition metal including Zn in various forms as exhibitor a good bacteria-inhibitory potential (Hadjer et al. 2018; Lakshmi and Geetha 2016).

The most common Zn derivatives used as feed supplements are Zn sulphates. However, they possess low accessibility and high toxicity. Another possibility is Zn–amino chelates with higher bioavailability and stability, revealed an excellent safety profile with superior get-at-ability for absorption than ZnSO_4_ in intestinal microorganisms (Abendrot et al. 2020). Peptide-zinc complexes are novel organometallic compounds with a variety of bio-functional activities. It is commonly reported that peptide-zinc chelate can improve the bioavailability of intestinal zinc in the human body (Wang et al. 2014). Similarly, previous studies have suggested that peptide-zinc chelates have strong anti-proliferative ability for pathogenic bacteria and simultaneously low toxicity for human body (Donaghy et al. 2023). Despite many researches on the structure and intestinal absorption of peptide-zinc chelates, few studies were devoted to their antimicrobial activity and mechanism. For example, Lin et al. (2019) in their research article studied antibacterial properties of chelating peptides-zinc nanocomposite against *E. coli* and described that possible action of its mechanism may be explained by the pathway of damaging cell membrane structure and oxidative stress-mediated apoptosis.

So far existing studies documented that susceptibility to Zn among microorganisms and even within strains of individual species can be highly variable (Fontecha-Umaña et al. 2020; Surjawidjaja et al. 2004) and moreover, administration of different forms and quantity of Zn can cause significant differences in inhibitory and/or stimulatory effect. Therefore, the effectiveness of Zn application in various forms and concentration should be appropriately investigated before it is recommended routinely as an adjunct therapy for treatment of different bacterial caused specific diseases or as feed supplement to improve animal or human health status.

Our finding confirmed that Zn-sulphates and Zn-amino acid complexes exhibit doses and genus-based antibacterial activity. We can conclude that organic complexes inhibited *E. coli* (Zn-Bio, MIC ═ 0.125 mg/mL; Zn-Gly, MIC ═ 0.75 mg/mL; Zn-AMK, MIC ═ 1.5 mg/mL), *K. oxytoca* (Zn-Gly, MIC ═ 1.5 mg/mL; Zn-AMK, MIC ═ 3 mg/mL; Zn-Bio, MIC ═ 8 mg/mL) better than both inorganic ZnSO_4_ (with MIC values ═ 2–6 mg/mL) except for Zn-Bio in case of *K. oxytoca*. The opposite results were obtained for *P. aeruginosa* (MICs were ranged at higher levels from 8 to 10 mg/mL for organic Zn in comparison with inorganic Zn with MIC value 2 mg/mL). Both inorganic ZnSO_4_ substances demonstrated better or comparable antibacterial activities against *S. aureus* (MIC ═ 1–3 mg/mL) e contra organic complexes (MIC ═ 1.5–4 mg/mL). Authors Abendrot et al. (2020) described that all amino acid-based complexes used by them exhibit better antimicrobial properties against Gram-positive bacteria than Gram-negative microorganisms and they postulate that such differences may be caused by a different structure of bacterial cell membranes. Similar results were obtained by Aiyelabola et al. (2016) for Zn complex with aspartic acid and moreover copper and cobalt amino acid complexes exhibited lower growth inhibitory potential towards Gram-negative bacteria (Stănilă et al. 2011).

Here, we report also anti-biofilm properties of various Zn inorganic and organic forms. Regarding to possible mechanisms, some reports suggest that certain metal elements, probably through lipopolysaccharides lead to decreased surface coating and thus prevent biofilm formation (Ansari et al. 2014; Edhari et al. 2021), respectively some authors investigated and confirmed their role in the anti-quorum sensing activity (Naik and Kowshik 2014).

## Conclusion

Multidrug resistant and biofilm-forming bacterial strains have great importance for public and veterinary health because of their role in certain infectious diseases and a variety of device related infections. In our study, we found some interesting effects of different Zn(II) complexes against the several common biofilm-forming multidrug resistant bacteria. The experiments showed that tested Zn inorganic and organic complexes had antibacterial and anti-biofilm potential. Organic compounds (Zn-AMK and Zn-Gly) were more effective against bacterial growth in contrast to inorganic Zn(II) forms, except *P. aeruginosa*. Besides Zn-AMK, others organic and inorganic forms of Zn(II) caused predominantly statistically significant decrease of biofilm production. Current data highlights that Zn has great potential to be developed as antibacterial and anti-biofilm agents. Further antimicrobial studies and cytotoxicity tests on our zinc complexes will be the subject of our next study. Of course, in vivo studies would also help to fill the knowledge-gaps in this important field.

## Data Availability

The datasets generated during and/or analysed during the current study are available from the corresponding author on reasonable request.
